# Thin-film lithium niobate electro-optic terahertz wave detector

**DOI:** 10.1038/s41598-024-55156-9

**Published:** 2024-02-27

**Authors:** Ingrid Wilke, Jackson Monahan, Seyfollah Toroghi, Payam Rabiei, George Hine

**Affiliations:** 1 Department of Physics, Applied Physics, and Astronomy, Rensselaer Polytechnic Institute, Troy, NY 12180 USA; 2 Partow Technologies LLC, Vista, CA 92081 USA; 3 Oak Ridge National Laboratory, Oak Ridge, TN USA

**Keywords:** Optics and photonics, Optical physics, Terahertz optics

## Abstract

The design, fabrication, and validation of a thin-film lithium niobate on insulator (LNOI) electro-optic (EO) time-domain terahertz (THz) wave detector is reported. LNOI offers unprecedented properties for the EO detection of freely propagating THz wave radiation pulses and transient electric fields because of the large EO coefficient of the material, engineering of the velocity matching of the THz wave and optical wave, and much reduced detector size. The proof-of-concept device is realized using thin-film lithium niobate optical waveguides forming a Mach–Zehnder interferometer with interferometer arms electrically poled in opposite directions. THz waves are coupled effectively to the fully dielectric device from free space without using antennas or plasmonics. The detection of THz waves with frequencies up to 800 GHz is successfully demonstrated. The detector allows for the detection of THz frequency electric fields up to 4.6 MV/m. The observed frequency response of the device agrees well with theoretical predictions.

## Introduction

Free-space electro-optic sampling of sub-picosecond THz frequency electromagnetic radiation pulses^1–3^ is highly important for time-domain THz wave spectroscopy^4^, time-domain THz wave imaging^5^, photonic time stretch measurements^6^, near-field THz wave microscopy^7^, and time-domain THz quantum optics^8,9^. The measurement modalities require electro-optic detection schemes with 0.1–10 THz bandwidths, detection thresholds of ~ 1 V/cm for THz wave spectroscopy and imaging, and ~ MV/cm dynamic range for longitudinal electron bunch lengths measurements at accelerators and nonlinear THz wave spectroscopy. Moreover, electro-optic measurements of radio-frequency (RF), millimeter (mm) and THz frequency electric fields are essential in fields such as electron beam diagnostics at accelerators^10,11^, plasma physics^12^, biomedical sensing^13^, laser radar^14^, microwave integrated circuit^15^ and antenna characterization^16^.

The linear electro-optic (EO) effect occurs in non-centrosymmetric crystals, wherein an applied electric field modifies the refractive index of the material, producing polarization and phase modulation, also known as the Pockels effect^17^. The EO effect occurs effectively instantaneously, enabling high temporal resolution. Additionally, all-dielectric EO sensors produce negligible distortion of the sampled electric field. The refractive index change of the EO crystal induced by THz frequency electric fields is probed by femtosecond (fs) near-infrared (nir) laser pulses time-synchronized with the freely propagating single-cycle sub-picosecond THz radiation pulses or transient electric fields. The sensitivity is dependent upon the Pockels coefficient of the EO crystal, the match of the velocities of THz and near-infrared wave traveling in the EO crystal, and their interaction length.

Lithium niobate (LN) is a versatile material for high frequency electric field sensing because of its large electro-optic material coefficients^17^, high transparency for visible and near-infrared waves (0.4–5 µm), and low absorption for RF, mm and THz waves (< 10 THz)^18^. Tightly confined LN waveguides fabricated from thin-film lithium niobate on insulator (LNOI) enable unprecedented possibilities for the engineering of velocity matching, dispersion engineering, and quasi-phase matching^19,20^. Groundbreaking proof-of-concepts using the thin-film lithium niobate (TFLN) platform are for example high-speed EO modulators^21,22^, EO frequency comb generators^23^, and most recently THz waveform synthesis^24^.

In this work, time-resolved EO detection of freely propagating THz radiation pulses using photonic integrated circuits fabricated in thin-film lithium niobate on insulator is reported. The approach to EO THz wave detector design innovatively exploits and integrates progress in materials science of thin-film LNOI, microfabrication of photonic integrated circuits and commercial communication wavelengths fiber optics. As proof-of-concept an original thin-film LNOI electro-optic detector chip has been designed, fabricated, and characterized. Effective phase-sensitive detection of the electric fields of freely propagating sub-picosecond THz radiation pulses with frequencies up to 800 GHz is demonstrated using the prototype device.

State-of-the-art EO detection of THz frequency electric fields utilizes bulk EO crystals^25^. The sensitivity and bandwidth of the detectors are limited by the phase mismatch (related directly to the refractive index mismatch) between the near-infrared and THz wave electric fields within the EO crystal. LN (LiNbO_3_) is an EO crystal that exhibits strong linear EO modulation (electro-optic coefficient *r*_33_ = 30.8 pm/V)^17^. Bulk LN crystals exhibit an unfavorably high phase mismatch at terahertz and sub-terahertz frequencies (∆*n* = *n*_THz_ –*n*_opt_ = 4.39 for *f* = 0.1THz and λ_opt_ = 1550 nm)^18^, yielding poor signal-to-noise ratios (SNRs) when used for the detection of freely propagating THz radiation pulses^25^. Electric field sensitivities as low as 1 Vm^−1^ Hz^−1/2^ have been demonstrated, but bandwidths in LN EO detectors have been limited due to the inherent phase mismatch characteristic for bulk LN crystals^16^. For EO sampling at frequencies above 100 GHz, ZnTe and GaP offer much larger bandwidths (ZnTe: *fc* = 3 THz; GaP: *fc* = 7 THz) but are limited in sensitivity by lower EO coefficients than LN (ZnTe: *r*_41_ = 3.90 pm/V; GaP: *r*_41_ = 0.97 pm/V)^26^.

Compared to the state-of-the-art, LN (LiNbO_3_) is superior to ZnTe and GaP for electro-optic detection of electric fields because of its substantially larger electro-optic coefficient. Importantly, thin-film LNOI enables perfect phase-matching of the THz wave signal and the near-infrared wave by appropriately engineering the optical waveguide. Moreover, effective, and stable spatial alignment of the laser probe beam with the EO THz wave detector is greatly simplified by guiding and coupling the laser beam to and from the detector using polarization maintaining optical fibers. The size and weight of the EO THz wave detector are drastically reduced because the photonic integrated circuit replaces multiple bulk optical components (and their mechanical mounts and holders). Future, cost-effective wafer-scale manufacturing of thin film LNOI detector chips is realistically envisioned.

State-of-the art EO THz waves detectors using LN and/or photonic integration are represented by device concepts where the incident THz wave electric field is locally enhanced using plasmonic antennas^27–30^ and plasmonics^31^. Our research objective aims at advancing the state-of-the-art by the development of a photonic integrated, fully dielectric EO sensor. The device is important and needed for non-invasive measurements of RF/millimeter/THz frequency electric fields and waves in environments where the presence of metal structures deposited on the LN may distort the electric field pattern to be examined.

## Results

### Photonic integrated circuit

**Figure 1:**
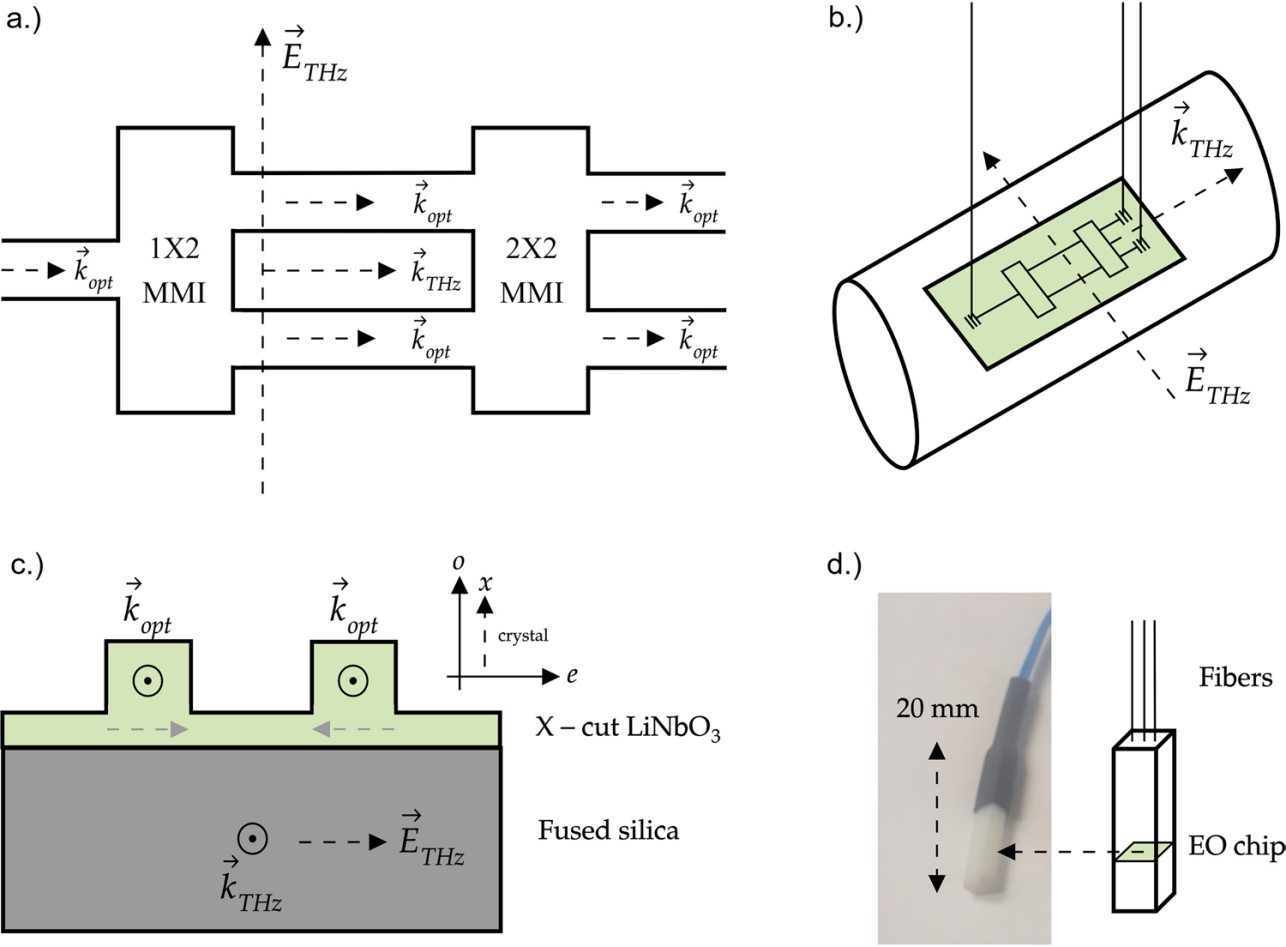
(**a**) Schematic top view of the thin film-lithium niobate (LN) photonic integrated circuit (PIC). THz waves (wave vector **k**_THz_) travel parallel to the two arms of the Mach–Zehnder interferometer (MZI) and parallel to the optical probe waves (wave vector **k**_opt_). The THz wave electric field E_THz_ is oriented parallel to the plane formed by the two arms of the MZI. (**b**) For measurements, the thin film LN electro-optic (EO) THz wave sensor chip with an active area of ≈ 10 µm (arm separation) × 600 µm (arm lengths) is placed next to or in the vicinity of the THz radiation beam. The THz radiation beam with beam diameters > 1 mm is schematically depicted as a cylinder. The drawing is not scaled. The optical fibers are oriented perpendicular to the surface plane of the EO sensor chip. Integrated gratings couple the optical probe laser light to and from the optical waveguides. (**c**) Schematic cross section view of the thin film LN waveguides on insulating fused silica. The LiNO_3_ crystal orientation is X-cut (in-plane extraordinary axis (**e**). The THz electric field is parallel to the extraordinary axis of LiNO_3_. The optical wave propagates as a TE mode in the waveguides with an in-plane optical electric field (not drawn). The intrinsic polarization of LiNO_3_ is indicated by dashed gray arrows. (**d**) Left: Photograph of a packaged sensor in its plastic housing with length scale indicated. Right: Schematic illustrating the location of the EO microchip within the plastic housing.

The thin-film LNOI electro-optic THz wave sensor design is illustrated in Fig. 1. It consists of a Mach–Zehnder (MZI) interferometer section (Fig. 1a) and one input and two output grating couplers (Fig. 1b). In the Mach–Zehnder interferometer section, the fiber-coupled light is divided between two arms using 1 × 2 MMI coupler. One MZI arm is poled to reverse the direction of spontaneous polarization of the lithium niobate crystal (Fig. 1c). Hence, for one MZI arm the refractive index increases for a given electric field while it will decrease for the same electric field in the other arm. Consequently, the laser light passing through the MZI is experiencing a phase shift of + $\Delta \varphi$ in one arm and a phase shift of − $\Delta \varphi$ in the other arm. THz wave is coupled to the MZI EO sensor from free space, the laser probe pulses are coupled to and from the electro-optic sensor chip using polarization maintaining fibers, which are oriented perpendicular to the sensor chip surface. The current device is made from 600 nm lithium niobate on a 500 µm fused silica substrate and operates at 1550 nm wavelengths. The output MMI 2×2 combines these two-phase modulated signals and produces an intensity-modulated signal.

The sensor is fabricated in X-cut LiNO_3_ where the extraordinary axis is in-plane, parallel to the surface of the sensor chip (Fig. 1c). The laser probe light travels in the optical waveguide as a TE mode with the electric field of the laser light oriented parallel to the surface. The electric field of the THz wave is parallel to the extraordinary axis. Both, the THz wave and the optical wave propagate collinearly. For this arrangement, the output of the MZI is described by (1)^17,32^:

1 $$\Delta \varphi \left( {\omega_{RF} } \right) = \frac{{\omega_{opt} }}{c} T_{RF} \left( {\omega_{RF} } \right) n_{e}^{3} r _{33} E_{THz} l = \frac{2\pi }{\lambda } T_{RF} \left( {\omega_{RF} } \right) n_{e}^{3} r _{33} l E_{THz}$$

In (2), c is the speed of light, ω_opt_ = (2πc/λ) is the probe laser light frequency and n_e_ = 2.15 the extraordinary index of refraction of LiNO_3_ at λ = 1550 nm^18^. The electro-optic coefficient of LiNO_3_ is r_33_ = 30.9 pm/V^17^. The THz wave electric field magnitude is E_THz_, the length of the interferometer arms is *l*. The transfer function T_RF_(ω_RF_) is defined as (2):

2 $$T_{RF} = \frac{{\sin \left( {\omega_{RF} \left( {n_{RF} - n_{opt} } \right)l/2c} \right)}}{{\omega_{RF} \left( {n_{RF} - n_{opt} } \right)l/2c}}$$

where *ω*_*R*_ is the THz wave frequency, *n*_*RF,*_ the THz frequency refractive index of SiO_2_, and n_opt_ the refractive index of LiNO_3_.

### Photonic integration

The miniature fiber-coupled thin-film LNOI EO THz wave detector is depicted in Fig. 1d. The introduction of microfabricated low-loss near-infrared waveguides, electrically poled LN, and fiber optics to the EO detection of transient THz frequency electric fields leads to high-level integration of functionalities previously supported by multiple bulk optical components. Quarter-wave plate/Wollaston prism, or alternatively an analyzer/polarizer pair are eliminated. The precise spatial alignment of the incident THz radiation and laser probe beams with the EO crystal is simplified using fiber-optics.

### Detector bandwidth engineering

Essential for the effective EO detection of THz frequency electric fields is the appropriate phase matching between the near-infrared laser probe pulse and THz wave electric field when both are traveling in the EO crystal. For an EO material with dispersion at near-infrared frequencies, phase matching is achieved when the phase velocity of THz wave is equal to the velocity of near-infrared pulse envelope (or the group velocity).

**Figure 2:**
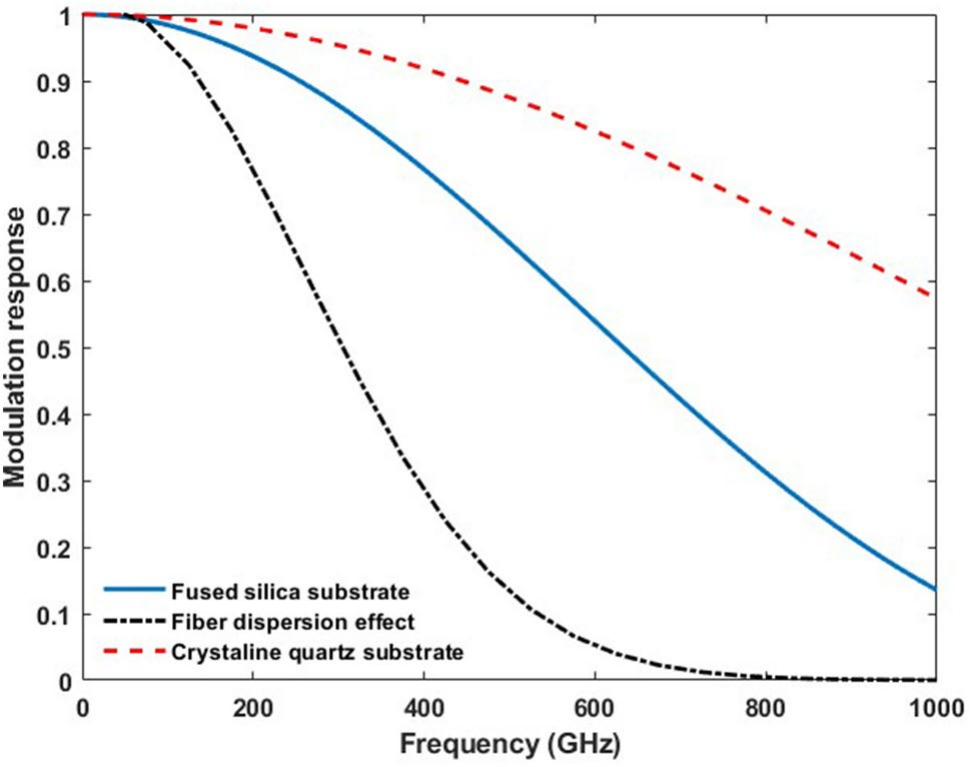
Calculated modulation response for thin-film LN waveguides on fused silica (blue) and crystalline quartz substrates (red) for a MZI type EO THz wave sensor with 600 µm path lengths. Modulation response (black dashed line) taking into account the dispersion of 40 fs probe laser pulses (λ = 1550 nm) in one meter of optical fiber for thin-film LN on fused silica and 600 µm electro-optic interaction length.

In thin film LiNO_3_ on fused silica (SiO_2_) substrates, the THz wave propagation speed is determined by the refractive index of SiO_2_ and is not affected by the LN thin film due to its very small volume compared to SiO_2_^22^. The index of refraction of fused silica is n_RF_ = 1.95 at 600 GHz^18^. This index is close to the group effective refractive index n_opt_ = 2.4 of the optical mode propagating in the thin film LN waveguide at λ = 1550 nm. The calculated normalized modulation response *|T*_*RF*_*|*^2^ of the device as a function of modulation frequency is shown in Fig. 2. The device tested in this work had an interaction length *l* of 600 µm and a predicted 3db bandwidths of 640 GHz.

### Observations

**Figure 3:**
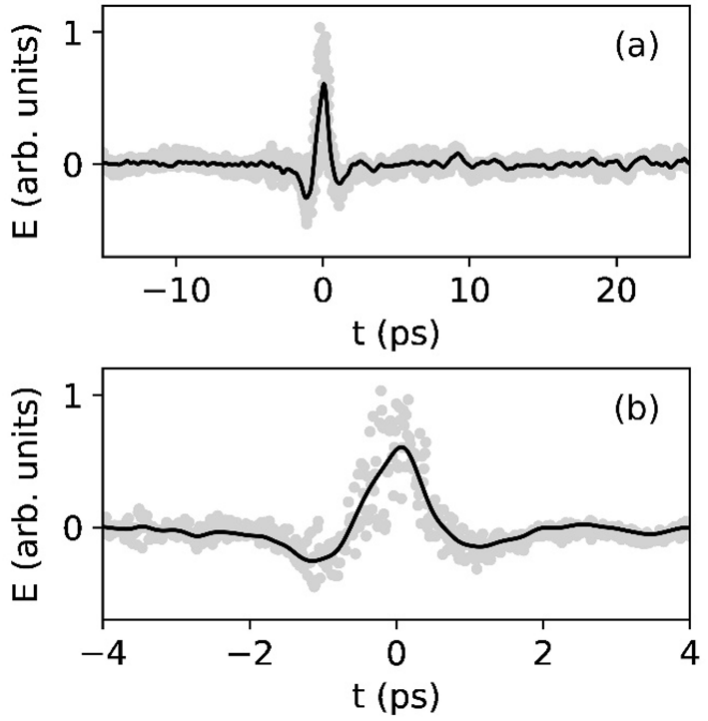
(**a**) Time-domain THz wave radiation pulse measured with the thin-film lithium niobate electro-optic THz wave sensor. Smoothed time-domain measurement (black solid line) overlaid on raw data (grey). (**b**) Close-up of the measured THz wave form.

The response of the thin film LNOI electro-optic THz wave detector to the incident THz wave radiation pulses measured in the time domain is illustrated in Fig. 3. The temporal profile of the signal agrees well with the typical profile of a THz wave radiation pulse generated from an OH1 crystal through optical rectification of femtosecond laser pulses. The duration of the measured THz radiation pulse is observed to be approximately 7 ps. The recorded signal changes from its largest negative value to its largest positive value on an observed timescale of ~ 1.4 ps.

### Signal-to-noise ratio

For THz-wave generation/detection with amplified laser systems, emitting 200 µJ pulses at a 1 kHz laser pulse repetition rate as in our experiment, state-of-the-art data acquisition is performed at the single shot (single laser pulse) level. The time-domain THz wave plotted in Fig. 3a represents the average of N = 16 THz waves each generated by an individual laser pulse. The signal-to-noise ratio (SNR) of our measurement as illustrated in Fig. 3 is typical for single-shot data acquisition and determined by the laser pulse to laser pulse energy fluctuations. This leads to pulse to pulse fluctuations of the THz wave electric field strength during THz wave generation at the OH1 crystal. The observed SNR is intrinsic to the detector. Future averaging of more waveforms will improve the signal-to-noise ratio as $SNR \approx \sqrt N$ with N representing the number of individually recorded waveforms. Linear photodetector arrays with spectral sensitivity between 350 and 5000 nm and frame acquisition rates of 10^6^–10^7^ frames per second for electro-optic decoding are documented in literature^33,34^. With this type of instrumentation the SNR can be improved to SNR $\approx$ 10^4^.

### Sensor bandwidth

**Figure 4:**
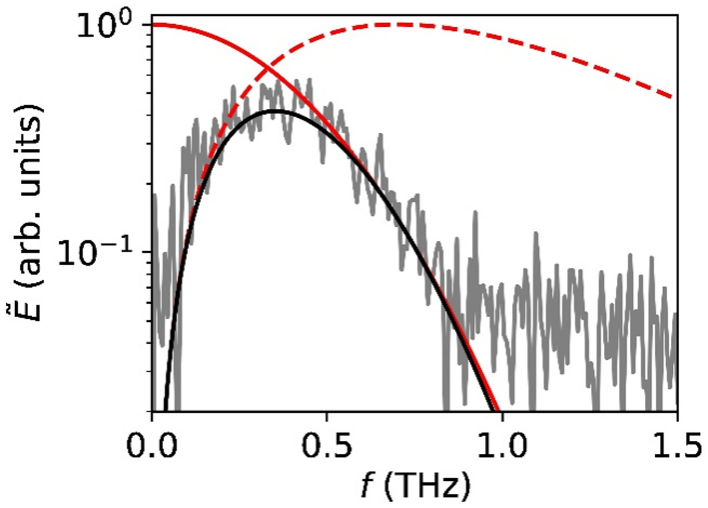
Thin-film lithium niobate electro-optic THz wave sensor frequency response (grey solid line) in comparison with the THz wave spectrum emitted by the OH1 THz wave radiation source (red dashed line). The calculated bandwidth of the thin-film LNOI modulator with an EO interaction length *l* = 600 µm for n_RF_ = 1.95 (fused silica) and n_opt_ = 2.4 and taking into account fiber dispersion of the 40 fs probe laser pulses is represented by the black solid line.

The Fourier spectrum of the signal recorded in the time-domain is displayed in Fig. 4. The observed profile of the spectrum is characteristic of a THz radiation pulse generated by optical rectification. The lowest and highest observed frequencies are approximately 100 GHz and 800 GHz respectively.

In Fig. 4, the frequency response of the thin film LNOI electro-optic detector is compared to the frequency spectrum of the THz wave source and the modulator bandwidth calculated according Eq. (2) for the MZI. The measured frequency response of the thin film LNOI electro-optic THz wave detector agrees well with the predicted low and high frequency limits of the modulator response. At high frequencies (> 500 GHz), an attenuation of the measured detector response in comparison with the calculated response is observed.

The observed high-frequency attenuation of the detection bandwidth compared to theoretical predictions is explained by the dispersion of the 40 fs laser pulses in the optical fiber. Fiber dispersion reduces the 3 dB bandwidths to 310 GHz (Fig. 2). However, fiber dispersion of the fs probe laser pulses can be effectively mitigated by using dispersion compensated optical fibers. The observed attenuated response of the device at low frequencies (< 250 GHz) in comparison with theoretical predictions, is typical for time-domain THz wave detectors and caused by geometrical aperture effects limiting the effective collection of low-frequency waves^35^.

The effect of THz wave and optical wave index mismatch (∆n = n_RF_ − n_opt_) on the sensor detection bandwidth is directly understood from (2). The transfer function T_RF_ approaches unity as ∆n approaches zero. Thus, the index mismatch must be minimized to maximize the detection bandwidth. This can be achieved by tuning the waveguide geometry to adjust n_opt_^36^, the addition of cladding layers to adjust n_RF_^21^, or using a different substrate material. As illustrated in Fig. 2, replacing fused silica with crystalline quartz will increase the 3 dB sensor bandwidths from 640 GHz (fused silica) to above 1 THz (crystalline quartz).

### Sensitivity

Equation (2) can be expressed using the half-wave electric field E_π_:

3 $$\Delta \varphi = \pi \frac{{E_{THz} }}{{E_{\pi } }} \,\,\,{\text{with}}\,\,\,\,E_{\pi } = \frac{\lambda }{{2 T_{RF} n_{e}^{3} r _{33} l}}$$

According to (3) the half-wave electric field is estimated to be E_π_ = 4.2 × 10^6^ V/m for T_RF_(ω_RF_) = 1.

The half-wave electric field E_π_ represents the upper limit of the dynamic range for THz frequency electric field sensing for our current device. The lower limit of the dynamic range is set by the smallest measurable phase shift $\Delta \varphi$. This depends on the approach to data acquisition. Using lock-in detection and a high repetition rate pulsed laser, it is possible to measure THz frequency electric field induced phase shifts of $\Delta \varphi \le 10^{ - 4} {\text{rad}}$. Following (3), the lower limit of the dynamic range of the device for the sensing of THz frequency electric field is estimated to be E_THz_ = 1.3 × 10^3^ V/m for E_π_ = 4.2 × 10^6^ V/m.

For identical EO interaction lengths *l* = 600 µm and modulation response T_RF_(ω_RF_), thin-film LN generates a higher phase shift $\Delta \varphi$ when compared to ZnTe and GaP because of its higher EO coefficient and lower index of refraction. This is illustrated by comparison of the half-wave fields E_π_ of the EO materials with the following ranking: E_π LN_ = 4.2 × 10^6^ V/m < E_π ZnTe_ = 1.6 × 10^7^ V/m < E_π GaP_ = 4.4 × 10^7^ V/m (for ZnTe and GaP the electro-optic coefficients and indices of refraction are r_41 ZnTe_ = 3.9pV/m, n_ZnTe_ = 2.73, and r_41 GaP_ = 0.97 pm/V, n_GaP_ = 3.1, respectively).

According to (1), for an applied THz electric field of magnitude E_THz_, the phase shift is determined by the laser probe wavelength λ, the transfer function T_RF_(ω_RF_), and the length of the MZI arm *l*. The phase shift can be increased by increasing the interaction lengths *l*, and reducing the laser probe wavelength λ. Inherently, minimizing the transfer function T_RF_(ω_RF_) will also maximize the device sensitivity. Thus, the signal–noise-ratio and the detection bandwidth are simultaneously improved by reducing the index mismatch ∆n = n_RF_ − n_opt_.

The sensitivity of the device is also influenced by the alignment of the propagation vectors **k**_THz_ and **k**_opt_ (Fig. 1 (b)) because of the small active area (10 µm arm separation × 600 µm arm lengths) of the device. If **E**_THz_ is not parallel to the in-plane extraordinary axis of LN, the applied THz frequency electric field **E**_THz_ still interacts with the TE mode of the waveguide but the *r*_33_ coefficient no longer contributes at full strength and the phase shift $\Delta \varphi$ is reduced. For an angle α > 0 between propagation vectors **k**_THz_ and **k**_opt_, the EO perturbation (EOP) under rotation for MZI type EO sensors made from X-cut LN is described by^37^:

4 $$EOP = \left( {\cos \alpha } \right)^{3} + \left( {\frac{{\varepsilon_{22} }}{{\varepsilon_{33} }}} \right)^{2} \left( {\frac{{r_{22} }}{{r_{33} }} \sin \alpha + \frac{{r_{23} }}{{r_{33} }}\cos \alpha } \right)\left( {\sin \alpha } \right)^{2}$$

**Figure 5:**
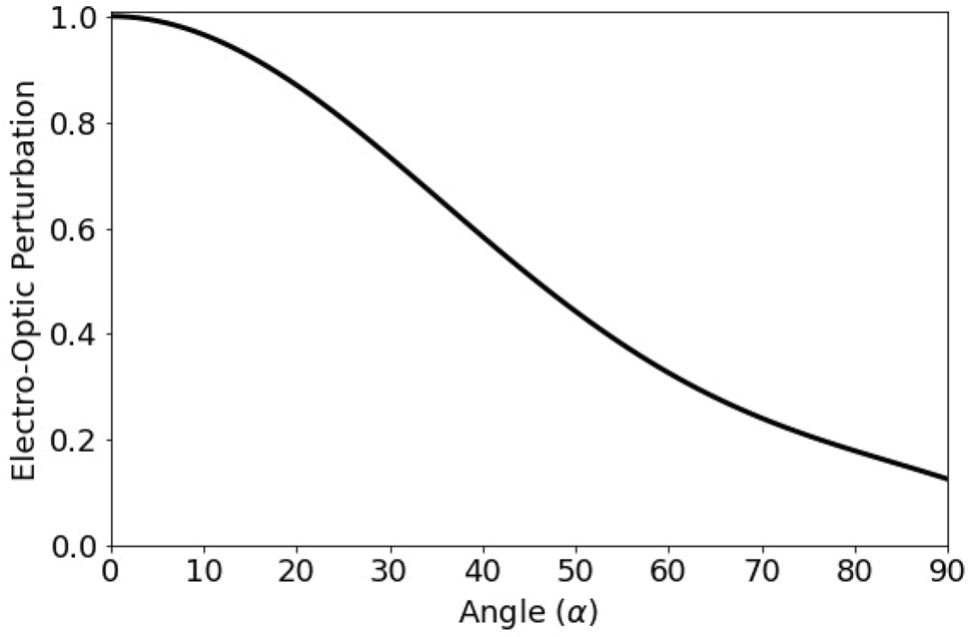
Electro-optic perturbation under rotation of X-cut LN (in-plane extraordinary axis) calculated according to ref.^36^. If the THz wave (**k**_THz_) does not propagate parallel to the guided TE optical mode (**k**_opt_), with α the angle between **k**_THz_ and **k**_opt_,_,_ the phase shift $\Delta \varphi$ induced by the THz wave electric **E**_THz_ through the Pockels effect is reduced.

For example, for an angle α = 10° between **k**_THz_ and **k**_opt_, the EO pertubation under rotation is EOP = 0.97 and the sensitivity is reduced by 3% compared to perfect alignment (Fig. 5). The estimate is made using LN material dielectric properties ^v^ε_11_ = ε_22_ = 4.889, ε_33_ = 4.569, r_22_ = 3.4pV/m, r_23_ = 8.6pV/m, and r_33_ = 30.9pV/m. EOP under rotation of the sensitivity can be can be eliminated by fabricating MZI type EO sensors made from Z-cut LN^37^.

### Conclusions

In summary, the design and fabrication of a photonic integrated circuit fabricated in thin-film lithium niobate on insulator technology is reported. Time-domain measurements of freely propagating single-cycle THz wave radiation pulses have been successfully performed using the Mach–Zehnder modulator device. THz waves are coupled to the fully-dielectric EO microchip detector from free space without the need of metallic antennas and plasmonic structures. The measured bandwidth of the prototype device agrees with calculations of the modulator response according to theory. The demonstrated proof-of-concept is an important step towards thin-film LNOI THz wave measurement systems for a broad range of users in science and industry.

## Methods

### Device fabrication

The thin-film LNOI wafer is fabricated by transferring a thin layer of crystalline lithium niobate onto a fused silica substrate using the crystal ion slicing method. For this purpose, a bulk LN crystal is ion implanted and bonded to the fused silica substrate. A subsequent heating process transfers a thin layer of lithium niobate to the fused silica substrate. The transferred crystalline thin film of lithium niobate has optical and electro-optical properties identical to the bulk LN crystals. After producing thin-film LN on fused silica, gold/chromium electrodes are deposited onto the wafer for the subsequent poling process. In order to reverse the direction of spontaneous electric polarization in one arm of the Mach–Zehnder modulator, the device is immersed in silicone oil and an electric field higher than the coercive field of lithium niobate (~ 22 kV/mm) is applied to the electrodes. Following electrode removal, the optical circuit of the EO THz wave sensor is patterned using e-beam lithography and is formed by dry etching of the lithium niobate layer. Fiber-optic v-groove arrays are subsequently aligned and attached to the device to achieve fiber-coupling. The device is inserted into a plastic housing for the protection of the optical fibers (Fig. 1d). The measured optical insertion loss of the device is − 13 dB at the operating wavelength λ = 1550 nm. More details of the fabrication process and device structures are explained in references^38–41^.

The basic functionality of the LNOI electro-optic electric field sensor was tested and confirmed by measuring the sensor response to electric fields oscillating at 100 kHz. The sensitivity of the detector was measured to be 2.2 Vm^−1^ Hz^−1/2^. Details of the test have been described elsewhere^41^.

### Experimental arrangements

**Figure 6:**
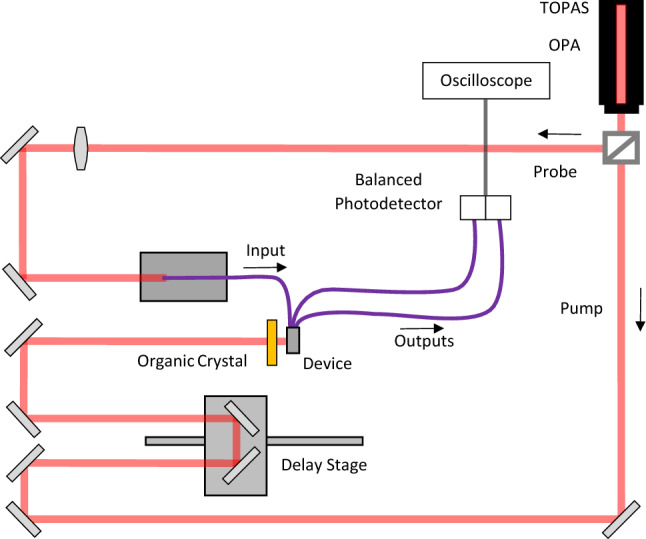
Experimental arrangements for THz radiation pulse generation and THz wave electro-optic detector testing.

Thin-film LNOI electro-optic detector characterization at THz frequencies was performed using two ultrashort 1550 nm laser pulses as depicted in Fig. 6. The laser probe pulse was free-space coupled into the Corning PANDA polarization maintaining single mode input fiber of the device using a fiber launch stage. The laser pump pulse was routed to an organic crystal (OH1) for THz wave generation by optical rectification.

The ultrashort 1550 nm laser pulses were generated by a Light Conversion TOPAS Prime optical parametric amplifier (OPA) pump with a Coherent Astrella Ti:Sapphire regenerative amplifier, operated at 1 kHz. The laser pulses emitted by the OPA at a wavelength of 1550 nm have 200 µJ of energy and a pulse length of 40 fs. The laser beam is split at a ratio of 7:1 at a variable polarizing beamsplitter with the p-polarized (horizontal) pump beam traveling through a variable delay line to the organic crystal for THz wave generation, and the s-polarized (vertical) probe beam traveling to the fiber launch stage. The OH1 crystal generates a THz bandwidth radiation pulse due to optical rectification of the laser pump pulse. Technical details of the THz radiation pulse generation are described in-depth in^42^.

Subsequently, the generated THz radiation pulse propagated through a high density polyethylene (HDPE) filter. For testing, the EO LNOI THz wave sensor is positioned 5 mm downstream from the HDPE filter.

Coupling the laser probe pulse into the input fiber of the detector device was achieved by overfilling the fiber entrance face with the unattenuated probe. This, along with an f = 100 mm focal length lens placed in the probe beam line mitigates the effects of pointing drift in the regenerative amplifier and provides a consistent probe fluence as measured at the outputs of the device. The possibility of a cladding mode is considered negligible as it will not transfer efficiently into the device due to the 1 × 2 MMI coupler.

The frequency modulated probe pulses are coupled from the MZ modulator arms into two Corning PANDA polarization preserving optical fibers which are directed towards a Thorlabs High-Speed InGaAs balanced photodetector (PDB230C). The internally amplified PDB230C has a transimpedance gain of 24.5 V/A for a 50 $\Omega$ load and a 3 dB RF bandwidth of 100 MHz. A collimator on each fiber focused the laser light into each of the detector’s photodiodes. The difference signal, along with monitors of the photocurrents on the individual diodes were recorded on an oscilloscope, averaging N = 16 individual shots. Since the laser probe pulse is significantly shorter than the electronic timescales of the balanced photodetector, the shape of each trace is given by the impulse response of the detector, and having a magnitude proportional to the difference of the light fluences. Since this difference is proportional to the THz electric field strength, the integrated balanced trace gives an instantaneous measure of the THz field at a specific pump-probe delay. By varying this delay, the temporal profile of the THz electric field can be reconstructed.

## Data Availability

The datasets collected and analyzed during the current study are available from the corresponding author on reasonable request.
