# Author Correction: Thin-film lithium niobate electro-optic terahertz wave detector

**DOI:** 10.1038/s41598-024-62574-2

**Published:** 2024-05-27

**Authors:** Ingrid Wilke, Jackson Monahan, Seyfollah Toroghi, Payam Rabiei, George Hine

**Affiliations:** 1 Department of Physics, Applied Physics, and Astronomy, Rensselaer Polytechnic Institute, Troy, NY 12180 USA; 2 Partow Technologies LLC, Vista, CA 92081 USA; 3 Oak Ridge National Laboratory, Oak Ridge, TN USA

Correction to: *Scientific Reports* 10.1038/s41598-024-55156-9, published online 27 February 2024

The original version of this Article contained an error in Figure 1, panels a and b, where half of the photonic integrated circuit (PIC) diagram was omitted.

**Figure 1:**
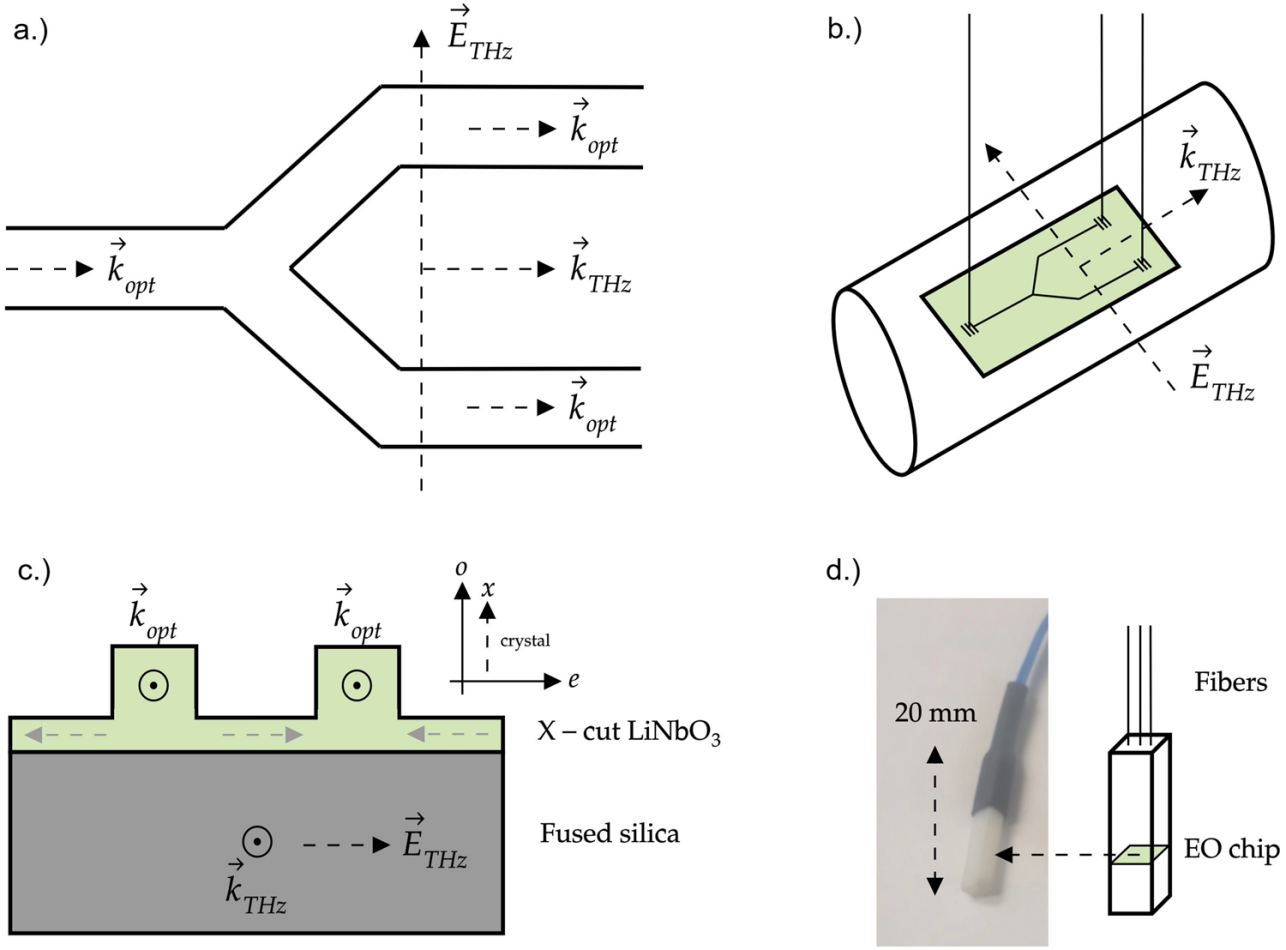
(**a**) Schematic top view of the thin film-lithium niobate (LN) photonic integrated circuit (PIC). THz waves (wave vector **k**_THz_) travel parallel to the two arms of the Mach–Zehnder interferometer (MZI) and parallel to the optical probe waves (wave vector **k**_opt_). The THz wave electric field E_THz_ is oriented parallel to the plane formed by the two arms of the MZI. (**b**) For measurements, the thin film LN electro-optic (EO) THz wave sensor chip with an active area of ≈ 10 µm (arm separation) × 600 µm (arm lengths) is placed next to or in the vicinity of the THz radiation beam. The THz radiation beam with beam diameters > 1 mm is schematically depicted as a cylinder. The drawing is not scaled. The optical fibers are oriented perpendicular to the surface plane of the EO sensor chip. Integrated gratings couple the optical probe laser light to and from the optical waveguides. (**c**) Schematic cross section view of the thin film LN waveguides on insulating fused silica. The LiNO_3_ crystal orientation is X-cut (in-plane extraordinary axis (**e**). The THz electric field is parallel to the extraordinary axis of LiNO_3_. The optical wave propagates as a TE mode in the waveguides with an in-plane optical electric field (not drawn). The intrinsic polarization of LiNO_3_ is indicated by dashed gray arrows. (**d**) Left: Photograph of a packaged sensor in its plastic housing with length scale indicated. Right: Schematic illustrating the location of the EO microchip within the plastic housing.

The original Figure 1 and its accompanying legend appear below.

In addition, in the Results section, under the subheading ‘Photonic integrated circuit’,

“THz wave is coupled to the MZI EO sensor from free space, the laser probe pulses are coupled to and from the electro-optic sensor chip using polarization maintaining fibers which are oriented perpendicular to the sensor chip surface. The current device is made from 600 nm lithium niobate on a 500 µm fused silica substrate and operates at 1550 nm wavelengths.”

now reads,

“THz wave is coupled to the MZI EO sensor from free space, the laser probe pulses are coupled to and from the electro-optic sensor chip using polarization maintaining fibers, which are oriented perpendicular to the sensor chip surface. The current device is made from 600 nm lithium niobate on a 500 µm fused silica substrate and operates at 1550 nm wavelengths. The output MMI 2×2 combines these two-phase modulated signals and produces an intensity-modulated signal.”

The original Article has been corrected.

